# *Borrelia bavariensis* in Questing *Ixodes ricinus* Ticks, United Kingdom

**DOI:** 10.3201/eid2910.230907

**Published:** 2023-10

**Authors:** Grace Plahe, Jessica L. Hall, David Johnson, Lucy Gilbert, Richard J. Birtles

**Affiliations:** University of Salford, Salford, UK (G. Plahe, R.J. Birtles);; University of Glasgow, Glasgow, Scotland, UK (J.L. Hall, L. Gilbert);; University of Manchester, Manchester, UK (D. Johnson)

**Keywords:** Lyme disease, Borrelia infections, *Borrelia bavariensis*, ticks, *Ixodes*, bacteria, vector-borne infections, United Kingdom, neuroborreliosis

## Abstract

We detected *Borrelia bavariensis* in *Ixodes ricinus* ticks collected near 2 towns in the United Kingdom. Human *B. bavariensis* infections have not been reported previously in the country, underscoring the value of tick surveillance to warn of emerging human disease. *B. bavariensis* should be considered in patients with suspected neuroborreliosis.

*Borrelia bavariensis* is one of several genospecies within the *B. burgdorferi* sensu lato complex to be associated with Lyme disease (*1*). *B. bavariensis* is widely distributed across Europe and Asia (*1*), although surveys of questing ticks in Europe indicate that *B. bavariensis* occurs at markedly lower prevalences than do other members of the *B. burgdorferi* s.l. complex (*2*). Of note, *B. bavariensis* appears to be overrepresented among *B. burgdorferi* s.l. clinical isolates in Europe (*3*), which has led to suggestions that it is more virulent than other members of the complex. Its potential enhanced pathogenicity, coupled with its specific association with neuroborreliosis (*3*), a profound manifestation of Lyme disease, has made *B. bavariensis* of particular medical concern.

During May–August 2022, we conducted questing tick surveys at 130 sites in and around 13 towns in the United Kingdom (Figure). We surveyed 10 sites around each town by conducting 15 blanket drag transects, 10 m × 1 m, per site. We identified all ticks collected morphologically as *Ixodes ricinus*, extracted DNA from each nymph and then incorporated each DNA extract separately as template in a real-time PCR to detect the presence of *B. burgdorferi* s.l. DNA (*4*). We processed 1 blank sample with every 5 nymphs to test for cross-contamination between samples; none of these blanks yielded a PCR product. In total, we found 91/1,311 nymphs (6.7%) to be infected with *B. burgdorferi* s.l. (Table). The positive samples were characterized by incorporating the DNA extracts into a conventional PCR that targeted the 5S/23S rDNA intergenic spacer region (*5*); the nucleotide base sequences were obtained by Sanger sequencing of both DNA strands. We used Geneious Prime software (https://www.geneious.com) to quality-check, collate, and analyze the sequence data. Of the 91 infected ticks, we successfully identified genospecies for 72. We encountered 5 genospecies: *B. afzelii, B. garinii, B. valaisiana, B. burgdorferi* sensu stricto, and *B. bavariensis* (Table). Given the high 5S/23S rDNA intergenic spacer region sequence similarity between strains of *B. garinii* and *B. bavariensis*, we incorporated the 4 DNA extracts suspected to be derived from *B. bavariensis* into a seminested PCR targeting an *ospA* fragment (*6*) and sequenced products of this reaction as described above. All 4 yielded unambiguous sequence data that were indistinguishable from one another. This sequence was identical to a 734bp *ospA* fragment of PBi, the type strain of *B. bavariensis* and <95% similar to *ospA* of representatives of other *Borrelia* genospecies (Genbank accession no. for the *ospA* sequence we obtained is OR208793).

**Figure:**
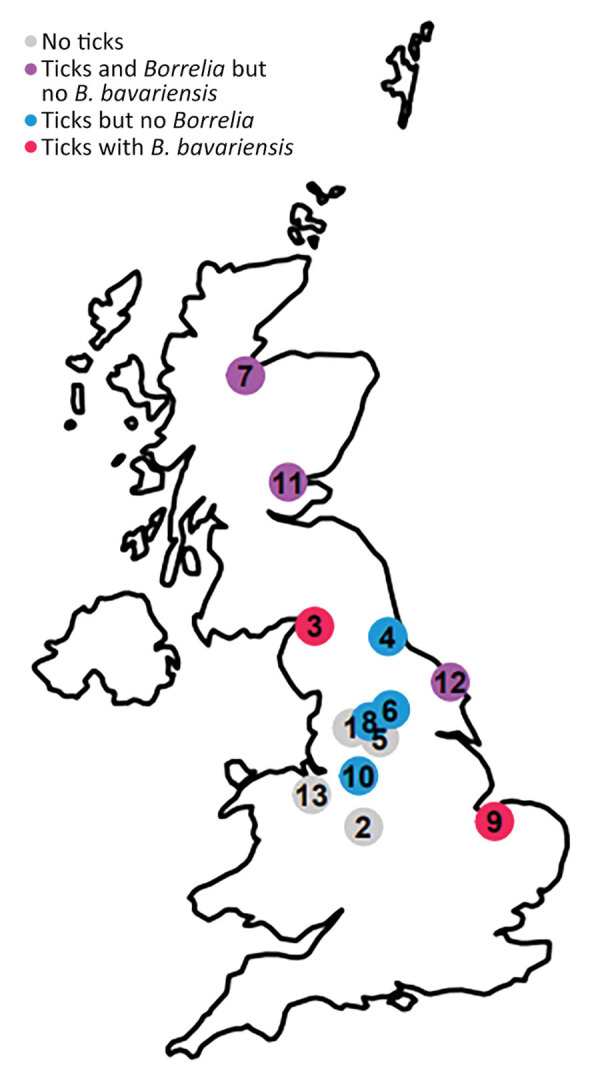
Relative locations of 13 towns where questing tick surveys were conducted in the United Kingdom to test for *Borrelia burgdorferi* sensu lato. The colors represent presence and absence of *Ixodes ricinus* ticks and their *Borrelia* infection status. The numbers correspond to the locations listed in the Table.

**Table:** Prevalence of *Borrelia burgdorferi* sensu lato in *Ixodes ricinus* tick nymphs sampled in 13 towns, United Kingdom*

Location	*I. ricinus* nymphs collected	*B.* *burgdorferi* s.l., no. (%)	*Borrelia *genospecies, no. (%), n = 91					
			*B. afzelii*	*B. bavariensis*	*B. burgdorferi s.s. *	*B. garinii *	*B. valaisiana*	Not identified
1 Burnley	0	0	0	0	0	0	0	0
2 Cannock	0	0	0	0	0	0	0	0
3 Carlisle	196	10 (5.1)	4 (40)	3 (30.0)	0	0	2 (20.0)	1 (10.0)
4 Durham	2	0	0	0	0	0	0	0
5 Halifax	0	0	0	0	0	0	0	0
6 Harrogate	40	0	0	0	0	0	0	0
7 Inverness	472	47 (10.0)	18 (38.3)	0	14 (29.8)	5 (10.6)	1 (2.1)	9 (19.1)
8 Keighley	7	0	0	0	0	0	0	0
9 Kings Lynn	59	1 (1.7)	0	1 (100.0)	0	0	0	0
10 Macclesfield	1	0	0	0	0	0	0	0
11 Perth	369	24 (6.5)	9 (37.5)	0	1 (4.2)	4 (16.7)	2 (8.3)	8 (33.3)
12 Scarborough	206	9 (4.4)	1 (11.1)	0	0	5 (55.6)	2 (22.2)	1 (11.1)
13 Wrexham	0	0	0	0	0	0	0	0
Total (%)	1,352	91 (6.7)	32 (35.1)	4 (4.4)	15 (16.5)	14 (15.4)	7 (7.7)	19 (20.9)

*Colors correspond to those used in the Figure. Prevalences for genospecies are in relation to the total infected ticks found in each town.

Our findings confirm the presence of *B. bavariensis* in questing *I. ricinus* nymphs in the United Kingdom. *B. bavariensis*–infected ticks were encountered at 2/130 sites surveyed, 1 near Kings Lynn and 1 near Carlisle, ≈325 km apart, which suggested a broad distribution. Encountering *B. bavariensis* is not entirely unexpected, given the pathogen’s wide distribution in temperate regions of the northern hemisphere and its adaptation to woodland rodents and hedgehogs (*7*), all of which are common across the United Kingdom. That earlier surveys in the United Kingdom have not encountered the species may reflect its relatively low prevalence in ticks and the patchiness of its distribution reported in continental Europe (*2*). In addition, methods used for delineating *Borrelia* genospecies may have lacked sensitivity. A previous study highlighted problems differentiating between *B. bavariensis* and *B. garinii* based on comparative analysis of 5S‐23S rRNA intergenic spacer region sequences; that approach is perhaps the most widely adopted across Europe and beyond (*8*).

Given the low number of *B. bavariensis* ticks encountered in the United Kingdom so far, drawing robust conclusions about the ecology of the genospecies is premature. Of interest, at the Carlisle site where we encountered *B. bavariensis*, 6/82 ticks tested from that site were infected with *B. burgdorferi* s.l. and 3 (50%) of those infected ticks carried *B. bavariensis*. *B. bavariensis* was not encountered in 113 ticks tested from the 3 other sites around Carlisle at which ticks were found. Those observations suggest that, even on a local scale, the occurrence of *B. bavariensis* is patchy, but enzootic hot spots may exist.

*B. bavariensis* is an addition to the list of zoonotic pathogens including *Anaplasma phagocytophilum*, *B. miyamotoi*, *Rickettsia helvetica*, and *Spiroplasma ixodetis* that are known to exist in UK ticks (*9*) but have yet to be reported in confirmed autochthonous cases in patients in the country. The recent confirmation of locally acquired encephalitis caused by tick-borne encephalitis virus (*10*) exemplifies the value of tick surveillance as an early warning of emerging human infections. Medical practitioners managing patients with suspected neuroborreliosis in the United Kingdom should now consider *B. bavariensis* as a potential infecting pathogen.
